# An Enzymatic and Proteomic Analysis of *Panus lecomtei* during Biodegradation of Gossypol in Cottonseed

**DOI:** 10.3390/jof10050321

**Published:** 2024-04-27

**Authors:** Clemente Batista Soares Neto, Taísa Godoy Gomes, Edivaldo Ximenes Ferreira Filho, Wagner Fontes, Carlos André Ornelas Ricart, João Ricardo Moreira de Almeida, Félix Gonçalves de Siqueira, Robert Neil Gerard Miller

**Affiliations:** 1 Laboratory of Microbiology, Department of Cell Biology, University of Brasília, Brasilia 70910-900, DF, Brazil; clementekeo@gmail.com (C.B.S.N.); taisa.godoy@gmail.com (T.G.G.); 2 Laboratory of Enzymology, Department of Cell Biology, University of Brasília, Brasilia 70910-900, DF, Brazil; eximenes@unb.br; 3 Laboratory of Protein Chemistry and Biochemistry, Department of Cell Biology, University of Brasília, Brasilia 70910-900, DF, Brazil; wagnerf@unb.br (W.F.); ricart@unb.br (C.A.O.R.); 4 Embrapa Agroenergia, Brasilia 70297-400, DF, Brazil; joao.almeida@embrapa.br

**Keywords:** *Panus lecomtei*, cotton, proteomics, biomass, gossypol, detoxification, animal feed

## Abstract

Cotton is an important plant-based protein. Cottonseed cake, a byproduct of the biodiesel industry, offers potential in animal supplementation, although the presence of the antinutritional sesquiterpenoid gossypol limits utilization. The macrofungus *Panus lecomtei* offers potential in detoxification of antinutritional factors. Through an enzymatic and proteomic analysis of *P. lecomtei* strain BRM044603, grown on crushed whole cottonseed contrasting in the presence of free gossypol (FG), this study investigated FG biodegradation over a 15-day cultivation period. Fungal growth reduced FG to levels at 100 μg/g, with a complex adaptive response observed, involving primary metabolism and activation of oxidative enzymes for metabolism of xenobiotics. Increasing activity of secreted laccases correlated with a reduction in FG, with enzyme fractions degrading synthetic gossypol to trace levels. A total of 143 and 49 differentially abundant proteins were observed across the two contrasting growth conditions after 6 and 12 days of cultivation, respectively, revealing a dynamic protein profile during FG degradation, initially related to constitutive metabolism, then later associated with responses to oxidative stress. The findings advance our understanding of the mechanisms involved in gossypol degradation and highlight the potential of *P. lecomtei* BRM044603 in cotton waste biotreatment, relevant for animal supplementation, sustainable resource utilization, and bioremediation.

## 1. Introduction

Currently, the world’s population is increasing by approximately 1.1% per year [1,2], with estimates of a population peak of 9.7 billion by 2050. As such, meeting the global demands for sustainable alternatives to animal-derived protein is a significant challenge [3]. Mitigation of climate change also requires rational use of the energy system to reduce greenhouse gas emissions. Limiting the global temperature increases to 1.5 °C above pre-industrial levels [4] will only be achieved if 60% of existing fossil oil and methane gas, and 90% of coal, all remain underground [5]. Although the transportation sector accounts for 65% of global refined oil consumption [6], this percentage can be significantly reduced with increased use of second-generation biodiesel, i.e., biodiesel produced from nonedible oilseeds [7].

Biodiesel is obtained through the chemical transesterification or esterification of fatty acids and triglycerides present in vegetable oils from oilseeds and animal fats [8]. As a renewable liquid bioenergy, biodiesel is biodegradable and does not contain toxic [9] compounds. Moreover, biodiesel is compatible with existing diesel engines and can be blended with diesel fuel in any proportion [9,10,11].

Although several oilseed crops offer potential in biodiesel production, 60% of global production is currently derived solely from soybean [12,13]. Besides the scale of agricultural land required and the resultant loss of biodiversity caused by monoculture production, soybean oil is edible and, by this characteristic, is defined as a 1st-generation biofuel, also competing with the food sector [7].

Given such limitations, cotton (*Gossypium hirsutum* L.), a species belonging to the Malvaceae family, offers considerable potential across different economic sectors. Apart from fibre employed in the textile industry, the oils contained in cottonseed cake, which is an abundant biomass released following fibre separation and cottonseed pressing, can then be converted into 2nd-generation biodiesel [14]. Given that cotton is ranked as the second-best source of plant-based protein, surpassed only by soybean [14,15], cottonseed cake can also be employed in animal supplementation. In the biodiesel industry, such generated byproducts can be as important as the oil itself and are appropriate for biorefinery models [16]. 

Although countries such as India, China, USA, Pakistan, and Brazil employ cottonseed cake as a protein source for animal feed formulations [17,18,19], the presence of antinutritional substances, such as condensed tannins [20,21], cyclopropenoid fatty acids [22], and, in particular, gossypol, can limit utilization [22,23].

Gossypol, a sesquiterpenoid aldehyde secondary metabolite, acts as a natural plant defence phytoalexin and is abundant in cottonseed, accumulating in aerial organ pigment glands and epidermal root tissues [22]. Its presence poses a challenge in animal feed since it can interfere with nutrient digestibility and/or absorption and is considered toxic, depending on the quantities consumed [18]. The molecule can occur in both free and bound forms [23,24,25], with the free form responsible for the toxic effects, especially in monogastric animals. According to the Food and Drug Administration (FDA), free gossypol in food products should not exceed 0.045%, equivalent to 450 ppm. However, for the European Food Safety Authority (EFSA), the maximum acceptable level in animal feed is 500 mg/kg or 500 ppm [26]. Ingestion of free gossypol in such animals can lead to reduced growth and weight gain, infertility due to the development of reproductive organ anomalies, impairment of liver function and respiration, inhibition of oxygen transport by erythrocytes, and can even result in heart failure and resultant animal mortality [27,28].

Given the high nutritional value of cottonseed cake and its potential as a substitute for conventional proteins, along with the increasing demand for second-generation biodiesel, efficient treatment to eliminate free gossypol is required. Biological pretreatment of cottonseed cake for animal consumption using macrofungi offers potential in the development of integrative and sustainable models where, in addition to enabling degradation of toxic components, treatment can also increase the nutritional value of residues [29]. For this, however, the identification of appropriate renewable biological agents capable of efficient degradation of free gossypol is essential.

*Panus lecomtei* is a macrofungus with potential in various biotechnological applications, ranging from detoxification of toxic and/or antinutritional factors to the production of industrial enzymes. In Brazil, the presence of this fungus has been documented extensively [30,31,32], where it also holds considerable significance within the cultural heritage of indigenous communities. In Yanomami villages such as Uauaris Xitei/Xidea, for example, the edible mushroom *P. lecomtei* is part of the local diet, where it is known as Shio-koni-amo or Kasikoirima [32]. Moreover, being an edible basidiomycete, it is also recognized as GRAS (Generally Recognized as Safe) for both humans and animals, ensuring its safe handling [33].

Previously, biological solid-state fermentation of cottonseed using *P. lecomtei* was shown to result in degradation of free gossypol to only trace levels [34]. Given the applicability of proteomics-based investigations into the identification, quantification, and evaluation of protein dynamics involved in different processes, this study characterized the secretome of *P. lecomtei* during growth on crushed cottonseed in the presence and absence of the antinutritional factor gossypol, enabling identification and quantification of proteins involved in the biodegradation of gossypol. Increased understanding of the mechanisms controlling gossypol degradation is essential to improve biorefinery models and efficient utilization of cottonseed and cake in sustainable biodiesel production.

## 2. Materials and Methods

### 2.1. Cottonseed and Fungal Strain

Processed and crushed cottonseed was provided by FARMOTEC^®^ (Buritis, MG, Brazil). Samples utilized in this study comprised untreated crushed whole cottonseed-containing gossypol (CWCS) and crushed whole cottonseed chemically treated with 2% Ca(OH)_2_ for removal of gossypol (CWCT). Detailed information regarding the bromatological composition of the substrates employed in this study have previously been described [34]. For the latter samples, the material was treated according to [34], who showed a 99.96% removal of free gossypol via this approach. The white rot basidiomycete *P. lecomtei* (strain BRM044603) was obtained from a local collection of microorganisms and microalgae at Embrapa Agroenergia [35] (under the Brazilian National System for the management of Genetic Heritage SISGEN registration number A06CD89), with molecular identification previously conducted by the group [34].

### 2.2. Cultivation of P. lecomtei on Crushed Whole Cottonseed

A total of 20 g (dry matter) of CWCS and CWCT, all previously moistened to 60% with sterile distilled water, was autoclaved at 121 °C for 15 min to remove any potential microbial contaminants on the residue. Whilst this physical treatment will also reduce free gossypol levels to approximately 15% of the original concentration, the expected concentration of free gossypol remaining in the cottonseed is in the region of 300 µg/g [34]. After cooling, a biological treatment of substrates was conducted in 250 mL Erlenmeyer flasks via inoculation with two 5 mm discs containing mycelial plugs of *P. lecomtei* BRM044603, previously grown for 7 days on potato dextrose agar. Cultures were then incubated for 15 days at 28 °C. All experiments were conducted in triplicate.

### 2.3. Quantification of Gossypol and Crude Enzyme Extracts

During the period of fungal colonization of cottonseed samples (CWCS and CWCT), free gossypol levels were measured every 72 h using the methodology proposed by [35]. For preparation of crude enzyme extracts for both untreated and treated samples, 100 mM sodium phosphate buffer at pH 6.5 was added at a ratio of 1:3 (m:v). The mixture was homogenized and incubated at 4 °C with agitation at 140 rpm for 40 min. Subsequently, the mixture was filtered and centrifuged for 10 min at 10,000 rpm at 4 °C. Total soluble proteins and enzymatic activities, including laccases, manganese peroxidase, lignin peroxidase, and proteases, were measured in crude enzyme extracts. All assays were performed in triplicate. Protein concentrations were determined using the Bradford method (BioRad, Hercules, CA, USA). Laccase activity (Lac, EC 1.10.3.2) was determined based on the oxidation of ABTS (2,2’-azino-bis-3-ethylbenzothiazoline-6-sulfonic acid) by mixing 180 μL of crude extract with 180 μL of 0.2 M sodium acetate buffer (pH 5, 25 °C). Oxidation of ABTS was monitored via spectrophotometry for 90 s at 25 °C, at a wavelength of 420 nm (Ԑ420: 36,000 L·mol^−1^cm^−1^) [36]. The assay of manganese peroxidase activity (MnP, EC 1.11.1.13) was carried out at 30 °C for 5 min using a 20 mM sodium succinate buffer at pH 4.5. MnP activity was determined by spectrophotometrically measuring the oxidation of phenol red at 610 nm (Ԑ10610 = 460 L·mol^−1^cm^−1^) [37]. The activity of lignin peroxidase (LiP, EC 1.11.1.14) was determined via the oxidation of veratryl alcohol in the presence of hydrogen peroxide, according to [38]. The reaction mixture comprised 500 μL of centrifuged enzymatic extract supernatant, 200 μL of 2 mM veratryl alcohol in 0.4 M sodium tartrate buffer (pH 3.0), and 200 μL of 2 mM hydrogen peroxide. Formation of veratraldehyde was monitored by measuring absorbance at 310 nm via UV-Vis spectrophotometry (SpectraMax M5, Molecular Devices, CA, USA) for 5 min, with readings taken every 10 s. Total proteolytic activity was determined at 37 °C using a modified method of [39], with 25 mg of azocasein and 5 mg of sodium bicarbonate per mL (pH 6.5) employed as substrates. A total of 500 μL of the enzymatic extract was mixed with 500 μL of azocasein. After 40 min at 37 °C, the reaction was stopped by adding 2.5 mL of 5% trichloroacetic acid. Absorbance of the centrifuged supernatant, diluted with 1 mL of 0.5 M KOH, was measured at 440 nm. An international enzyme unit (U) was defined as an enzyme quantity that produces 1 μM of product formed per minute. All experiments were carried out in triplicate.

### 2.4. Global Secretome Analysis

Cultivation of *P. lecomtei* BRM044603 on CWCS and CWCT was conducted as described above, with cultures incubated in a controlled temperature incubator at 28 °C. In order to obtain the proteomic profile of the fungal secretome, samples were collected 6 and 12 days after inoculation (DAI). Differentially abundant proteins were identified through comparison of data derived from cultivation on CWCS and CWCT.

### 2.5. Sample Preparation and Processing

Crude protein extract preparation was conducted following diluting CWCS and CWCT in chilled distilled water. For this, 15 g of substrate was diluted in 30 mL of distilled water in a 150 mL Erlenmeyer flask and the mixture homogenized on an orbital shaker at 5 °C for 40 min. Crude secreted enzyme extracts were filtered using a Buchner funnel with a 50% viscose and 50% polyester fabric. Extract samples were then desalted through tangential filtration using 5 Kda filters, assayed for crude protein concentration via the BCA method, and concentrations standardized to a final concentration of 100 µg/mL. All experiments were performed in triplicate. Samples were subsequently submitted to reduction with DTT, alkylation with iodoacetamide, and trypsin digestion. Tryptic peptides were desalted using C-18 microcolumns and quantified on a Qubit system [40,41]. Secretome aliquots (BRM044603-CWCS and BRM044603-CWCT), each containing 20 µg of total protein, were precipitated [41], dried under vacuum, and reconstituted in 150 µL of a solution containing 8 M urea, 7.5 M NaCl, 50 mM triethylammonium bicarbonate (TEAB), and 5 mM dithiothreitol (DTT), at pH 8.2, and incubated at 55 °C for 25 min. Iodoacetamide was added to a final concentration of 14 mM, followed by a 40 min incubation at 25 °C in darkness. DTT was then added to a final concentration of 10 mM, and the reaction mixture diluted five times in 25 mM TEAB at pH 7.9, with the addition of CaCl_2_ to a final concentration of 1 mM. Samples were digested with modified porcine trypsin (1 µg per 50 µg of total protein) at 37 °C, followed by the addition of trifluoroacetic acid (TFA) to a final concentration of 0.5% (*v*/*v*). Samples were then dried under vacuum, reconstituted in 0.1% (*v*/*v*) TFA, and desalted using StageTips filled with Empore C18 membranes [40]. Trypsic peptides were finally quantified via fluorometry (Qubit, Thermo Fisher Scientific, Waltham, MA, USA).

### 2.6. LC-MS/MS Analysis

Chromatographic and mass spectrometry analyses were performed as described by [42] and [43], with the following adaptations.

### 2.7. Chromatography

Peptides were injected into a Dionex chromatographic system (Dionex Ultimate 3000 RSLCnano UPLC, Thermo, Waltham, MA, USA), configured using a 3 cm × 100 µm trapping column with C18 particles (5 µm, 120 Å), connected in series with a 24 cm × 75 µm analytical column containing C18 particles (3 µm, 120 Å) (ReprosilPur, Dr. Maich GmbH, Ammerbuch, Germany). From a total of 1 µg injected into the column, samples were then subjected to a linear elution gradient using two solvents, namely solvent A (0.1% formic acid in water) and solvent B (0.1% formic acid in acetonitrile), starting from 2% B and reaching 35% B over a 155 min period.

### 2.8. Mass Spectrometry

The separated fractions via chromatography were directly eluted into the ionization source of an Orbitrap Elite mass spectrometer (Thermo, Waltham, MA, USA), which was configured for operation using a data-dependent acquisition (DDA) mode, where the MS1 spectra were acquired with a resolution of 120,000 and a m/z range of 300 to 1650. The 20 most intense ions above an intensity threshold of 3000 were subjected to fragmentation, generating MS2 spectra in the ion trap analyser through CID [44,45]. In order to favour the identification of less abundant peptides, reanalysis of previously fragmented ions was inhibited via dynamic exclusion [42].

### 2.9. Data Analysis

Obtained spectra were qualitatively and quantitatively analysed to identify both the total set of detectable proteins in the samples and to quantitatively evaluate proteins with significantly different relative abundance between conditions. Proteins originating from the different conditions were grouped according to pathways, GO terms, and interactions. The methods employed generally followed the strategy described in [42], with adaptations described below.

Qualitative Analysis: The complete set of spectra was analysed using the software Peaks, version 7.0 (BSI, USA), through a search against a database from the Uniprot repository, filtered for the order Polyporales, tax ID 5303, which contains the species *Panus lecomtei*, taxon ID 38810 (Polyporales—Panaceae—Panus—*Panus lecomtei*), added to a database filtered for the species *Gossypium hirsutum*, taxon ID 3635. The combined database was filtered for removal of redundant sequences using the software FASTAtools, version 1.2.0. A search was performed based on de novo sequencing and PSM (Peptide-Spectrum Matching), with a precursor mass tolerance of 10 ppm and fragment tolerance of 0.5 Da. Additionally, search modules were activated for modifications from the Unimod database based on fragmentation patterns, as well as point mutations [46].

Quantitative Analysis: Spectra were analysed using the software Progenesis QI for Proteomics (Waters, Milford, MA, USA) [47]. This involved aligning the chromatograms, quantification by area of the extracted peaks (XIC—extracted ion chromatogram), normalization, and statistical analysis (ANOVA) of MS1 events. For significantly different events (*p*-value < 0.05), MS2 spectra were identified using the software Peaks, version 11, employing the parameters described in the previous section.

### 2.10. Protein Grouping and Functional Inference

The database accession codes for the differentially abundant proteins were used to obtain GO terms associated to such proteins via the BioDBnet, BlastKoala, and Blast2GO platforms [48,49,50,51]. Proteins were also grouped based on their participation in signalling pathways, as well as on the basis of protein–protein interactions. Enrichment of GO terms, pathways, and interactions, as well as protein interaction clustering, was statistically evaluated using the Panther and String platforms, following ortholog mapping to *Saccharomyces cerevisiae* [52,53,54]. GO terms were also semantically grouped using the ReviGO system [55].

### 2.11. Partial Purification of the Crude Extract

Samples obtained from the crude enzyme extracts (ECs) were subjected to size exclusion chromatography according to the following methodology by [56] with adaptations. Samples were applied to a Superdex S-200 size exclusion column, coupled to an Akta Pure system (GE Healthcare), prior to dialysis in Tris HCl buffer at pH 7.0 containing 0.15 M NaCl. The column was equilibrated with 48 mL of the dialysis buffer at a flow rate of 0.5 mL/min. Protein injection and elution processes were carried out at a flow rate of 0.5 mL/min. Fractions with a peak absorbance at 280 nm were collected for the untreated CWCS as follows: S1, S2, S3, S4, S5, S6, S7, S8, and S9, and for the chemically treated CWCT sample as follows: S2, S3, S4, S5, S6, S7, and S8. The fractions obtained in this step were applied for analysis of synthetic gossypol degradation using the following parameters: 2 mL from each fraction were concentrated and dialyzed four times in sodium phosphate buffer (100 mM) at pH 6.5 using an Amicon system. Subsequently, 200 µL from each fraction were mixed with 2 µL of synthetic gossypol and incubated at 37 °C for 120 min at 240 rpm. From the results of this assay, 10 µL from each sample were diluted in 1 mL of sodium phosphate buffer (100 mM) at pH 6.5, and the residual free gossypol was quantified. Protein concentrations were determined via the Bradford method [57], employing a commercial quantification kit from BioRad (Hercules, CA, USA), in strict accordance with the manufacturer’s instructions and with measurements at A280. Laccase enzymatic assays were also performed on the samples, as described above.

## 3. Results

### 3.1. Degradation of Free Gossypol (FG)

**Figure 1:**
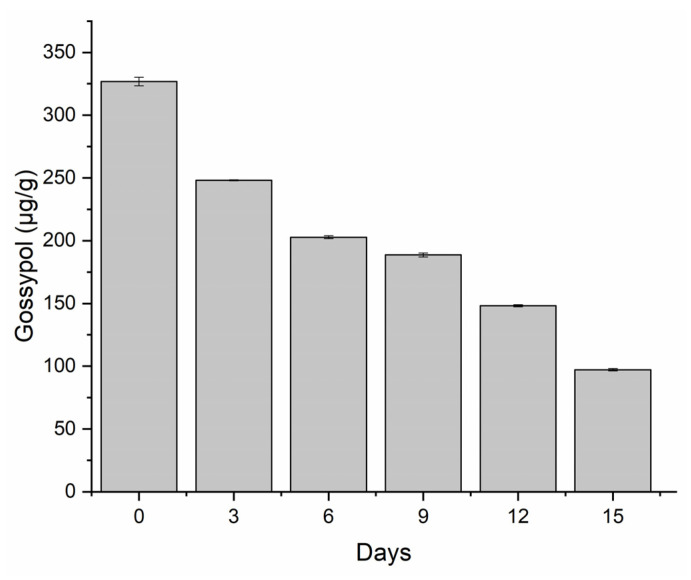
Degradation of free gossypol by the macrofungus *Panus lecomtei* during a 15-day cultivation period at 28 °C on crushed whole cottonseed with gossypol (CWCS).

*P. lecomtei* BRM044603 demonstrated a remarkable capacity for degradation of FG during the initial three days of growth on CWCS, with the degradation rate also increasing progressively over the cultivation period. After 15 days of growth, FG degradation reached a total of 70%, with a final concentration detected at only 100 µg/g (Figure 1). Analysis at each time point also revealed that the rate of FG degradation was not constant, with the highest variation observed between the 12th and 15th day of cultivation, during which period a reduction in FG of 34.46% was observed. An additional peak in degradation was also evident during the first six days of cultivation, with a reduction of 42.09% in FG levels.

### 3.2. Enzyme Profiling during Cultivation of P. lecomtei BRM044603

**Figure 2:**
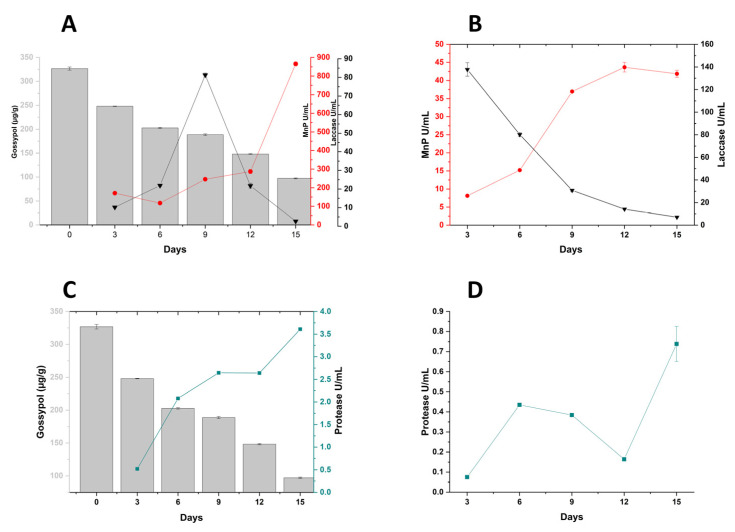
Enzymatic induction profile in *Panus lecomtei* during a 15-day cultivation period in the presence and absence of gossypol. (**A**) Activity of oxidative enzymes (Laccase [pH 5.0 at 25 °C] and MnP [pH 4.0 at 30 °C]) in the presence of gossypol (crushed whole cottonseed-CWCS); (**B**) activity of oxidative enzymes (Laccase [pH 5.0 at 25 °C] and MnP [pH 5.0 at 25 °C]) in the absence of gossypol (chemically treated crushed whole cottonseed-CWCT); (**C**) proteolytic activity [pH 6.5 at 37 °C] in the presence of gossypol (CWCS); and (**D**) proteolytic activity [pH 6.5 at 37 °C] in the absence of gossypol (CWCT).

Analysis of lignocellulolytic and proteolytic enzyme activities for *P. lecomtei* cultivated on substrates in the presence (CWCS) and absence (CWCT) of FG are summarized in Figure 2. These enzymes were selected for quantification based on their known proficiency in degrading a variety of xenobiotic compounds. Notably, among the enzymatic activities quantified (MnP, laccase, protease, and lignin peroxidase), only LiP remained undetected in all substrates evaluated throughout the 15-day period. The expression profiles for MnP and protease enzymes were similar in both growth conditions in the presence or absence of FG, suggesting that these enzymes are not induced by the toxic compound. A maximum activity of MnP was observed in CWCS at 869.79 U/mL after 15 days of cultivation, with the increase in enzyme activity correlating with cultivation period. MnP activity was also observed in CWCT, although at lower concentrations compared to those on CWCS. As observed previously, there was also an increase in enzyme activity over the cultivation period (Figure 2A,B). Protease activity also increased as a function of cultivation period, with enzyme profiles similar on both substrates (Figure 2C,D). When comparing data on CWCS with CWCT, however, higher rates were observed in the CWCS medium, with a maximum activity of 3.61 U/mL on the final day of cultivation (15 days), in contrast to a maximum value of 0.79 U/mL at the same time point on CWCT. Observed levels of proteases were considerably lower than those for the MnP oxidative enzymes throughout the examined time course and across both substrates.

In contrast to the increased activities of MnP and proteases over the cultivation period, laccase enzyme activity profiles were distinct to those of the other enzymes on both substrates (Figure 2A–D). Whist activities were observed across all time points investigated for both growth conditions, activity profiles differed between CWCS and CWCT. In the case of growth on CWCS, activities increased between 3 and 9 days (10.16 U/mL and 81.23 U/mL, respectively) with a subsequent decrease in production at 12 and 15 days (21.78 U/mL and 2.68 U/mL, respectively) (Figure 2A). These data indicated a potential involvement of laccases in the degradation of gossypol, with an early rise in activities correlating with a continued reduction in FG. Laccase activities on CWCT, by contrast, displayed a continuous decline over time, from 137.75 U/mL at 3 days to 7.06 U/mL at 15 days (Figure 2B).

Considering the enzyme activity changes over time during the growth conditions in the presence and absence of FG, together with the observed rate of FG degradation, 6 and 12 DAI were chosen as appropriate time points for investigation of the fungal secretome in relation to FG degradation.

### 3.3. Proteomic Analysis of Crude Extracts from P. lecomtei BRM044603 Cultivated in the Presence of Gossypol (CWCS) and in the Absence of Gossypol (CWCT) at 6 DAI and 12 DAI

**Figure 3:**
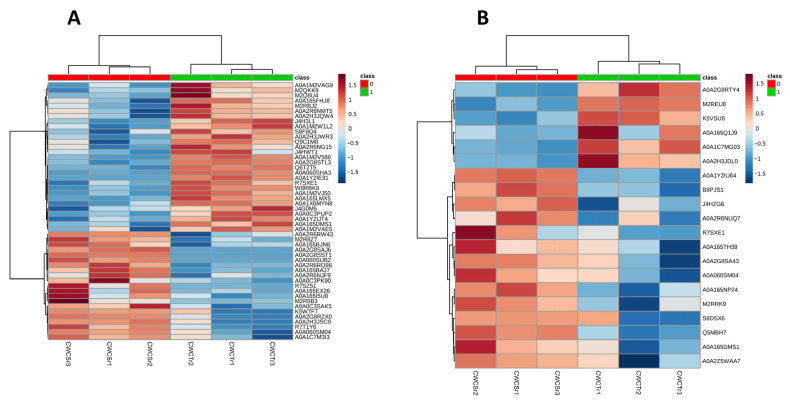
Heatmap of correlations between normalized abundancy profiles in *Panus lecomtei* considering two growth conditions (presence or absence of gossypol in cottonseed). (**A**): Set of the 50 proteins with the lowest p-values at 6 DAI (days after inoculation) and (**B**): set of the 20 proteins with the lowest p-values at 12 DAI.

**Figure 4:**
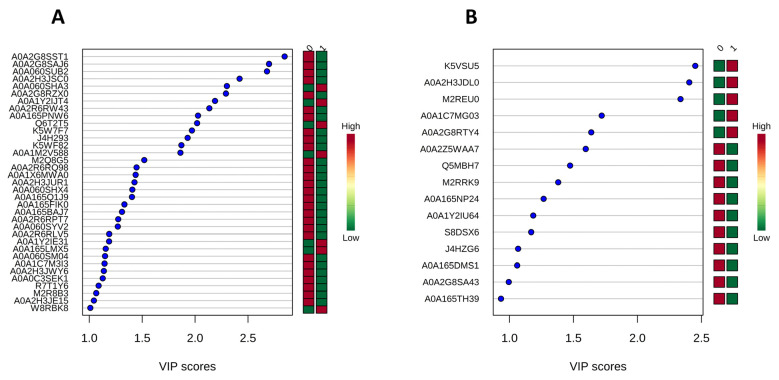
VIP scores of *Panus lecomtei* proteins that exhibit the highest discriminatory power between conditions (presence or absence of gossypol in cottonseed). (**A**): VIP scores for the 35 proteins identified at 6 DAI (days after inoculation) and (**B**): VIP scores for the 15 proteins identified at 12 DAI.

At 6 DAI, a total of 307 regulated proteins were identified in the crude extracts following growth of *P. lecomtei* under the two examined conditions. Contrasting protein profiles were observed between the treatments, with biological replicates clearly grouped together (Supplementary Figure S1). Among the regulated proteins, a total of 18 were present in the *Gossypium hirsutum* database tax ID 3635. By applying an additional stringent criterion (proteins identified with two or more peptides), a total of 143 regulated proteins were identified, none of which belong to *G. hirsutum*. The correlation map (heatmap) based on abundance profiles for the top 50 proteins at 6 DAI confirmed the distinct protein expression profiles between the examined conditions, with 21 proteins more abundant on CWCS and 29 more abundant on CWCT (Figure 3A). Although correlation analysis indicated a higher number of proteins in CWCT, Variable Importance Projection scores (VIP scores) identified 35 proteins with a higher discrimination between the tested conditions, of which 28 were more abundant in CWCS and only 7 in CWCT (Figure 4A). Additionally, 17 proteins were not identified in the database (unnamed/hypothetical). Noteworthy among the abundant proteins identified in CWCS were ribosomal proteins, aconitase, metalloproteinase, acetyl-CoA carboxylase, isocitrate dehydrogenase, reductase, and oxidoreductase. In contrast, in CWCT, six proteins were identified as abundant: glucanase, peroxidase, laccase, cytochrome b5, Mn(III)-dependent proteinase, and thioredoxin reductase. With regard to the results obtained at 12 DAI, 122 regulated proteins were identified, showing distinction between the conditions (Supplementary Figure S1). Only 4 of these proteins were found in the plant database (*G. hirsutum*), and applying the stringent criterion described above, a total of 49 regulated proteins were observed, none of which belonged to *G. hirsutum*. Compared to 6 DAI, both the number of abundant proteins and their abundance patterns were different. The correlation map (heatmap) for the top 20 most abundant proteins indicated a distinct protein profile between the analysed conditions, with 14 proteins being more abundant in CWCS and 6 in CWCT (Figure 3B). VIP score data identified 15 proteins that best discriminated between the conditions (Figure 4B), with 5 proteins showing higher abundance in CWCT: pyrophosphatase, dehydrogenase, ribosomal protein, and two hypothetical proteins. In contrast, 10 proteins were identified as best discriminating in CWCS, with noteworthy proteins including peroxidase, laccase, phosphoenolpyruvate carboxykinase, dienelactone hydrolase, isocitrate dehydrogenase, heat shock protein, glutathione reductase, and enolase. Interestingly, the peroxidase and laccase proteins identified at 12 DAI were more abundant on CWCS and differed from those identified at 6 DAI, which were more abundant on CWCT.

### 3.4. Functional Analysis of the Global Secretome of P. lecomtei BRM044603 via Gene Ontology (GO), String-Derived Protein–Protein Interaction Networks, and Kyoto Encyclopedia of Genes and Genomes (KEGG) Metabolic Pathway Mapping

**Figure 5:**
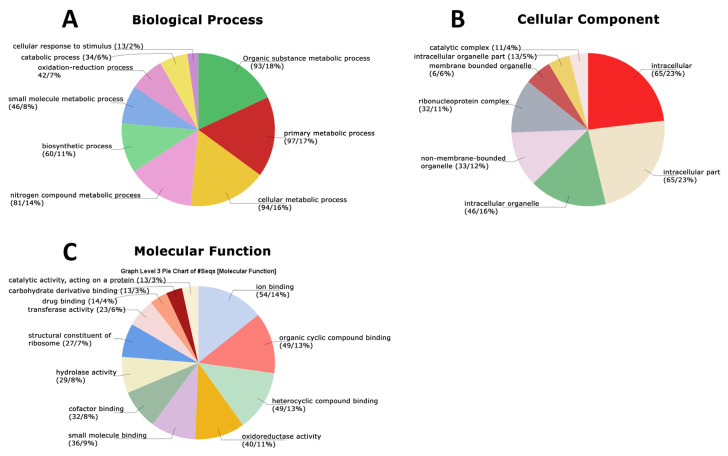
GO terms identified in protein secretome data at 6 days after inoculation (6 DAI) of *Panus lecomtei* considering two conditions: presence or absence of gossypol in cottonseed.

**Figure 6:**
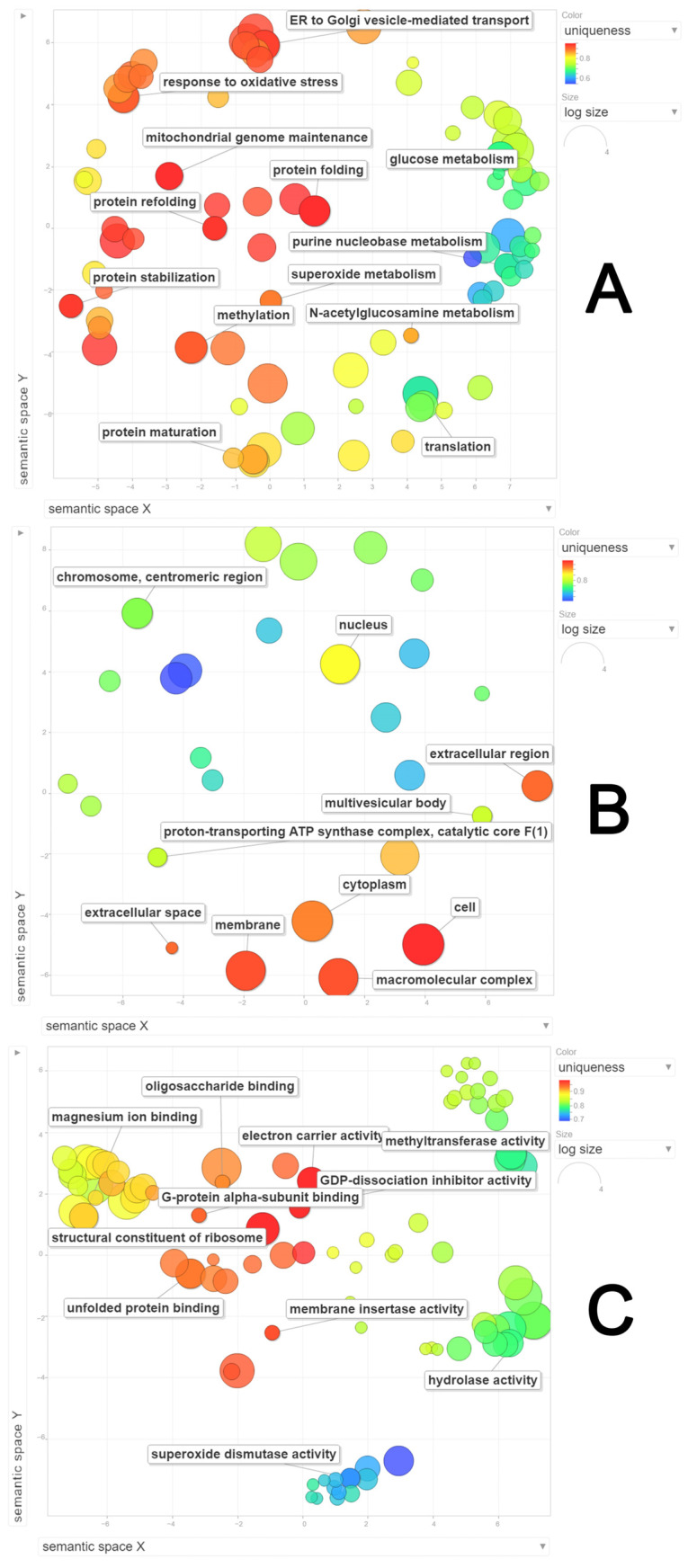
Semantic clustering of proteins identified at 6 days after inoculation (6 DAI) of *Panus lecomtei* considering two conditions: presence or absence of gossypol in cottonseed. (**A**) Biological process; (**B**) Cellular component; (**C**) Molecular function.

GO terms were identified from protein secretome data for both cultivation time points examined during growth in the presence and absence of FG. At 6 DAI, a total of nine biological process terms were detected: organic substance metabolic process (18%), primary metabolic process (17%), cellular metabolic process (16%), nitrogen-compound metabolic process (14%), biosynthetic process (11%), small-molecule metabolic process (8%), oxidation–reduction process (7%), catabolic process (6%), and cellular response to stimulus (2%) (Figure 5A). The semantic grouping of proteins according to biological processes revealed a total of 13 categories, with the greatest representation in glucose metabolism, ER-to-Golgi-vesicle-mediated transport, response to oxidative stress, glucose metabolism, and purine nucleobase metabolism, respectively (Figure 6A). The most frequent categories included methylation, protein folding, protein refolding, protein stabilization, protein translation, and protein maturation. With regard to cellular components, eight categories were identified: intracellular (23%), intracellular part (23%), intracellular organelle (16%), nonmembrane-bounded organelle (12%), ribonucleoprotein complex (11%), membrane-bounded organelle (6%), intracellular organelle part (5%), and catalytic complex (4%) (Figure 5B). Semantic grouping indicated 10 terms, with a higher representation of macromolecular complex, membrane, cytoplasm, cell, and nucleus (Figure 6B). For molecular functions, a total of 12 categories were identified: ion binding (14%), organic cyclic compound binding (13%), heterocyclic compound binding (13%), oxidoreductase activity (11%), small molecule binding (9%), cofactor binding (8%), hydrolase activity (8%), structural constituent of ribosome (7%), transferase activity (6%), drug binding (4%), carbohydrate derivative binding (3%), and catalytic activity acting on a protein (3%) (Figure 5C). Semantic grouping revealed 11 terms, with a higher representation of magnesium ion binding, hydrolase activity, superoxide dismutase activity, unfolded protein binding, methyltransferase activity, and oligosaccharide binding (Figure 6C).

**Figure 7:**
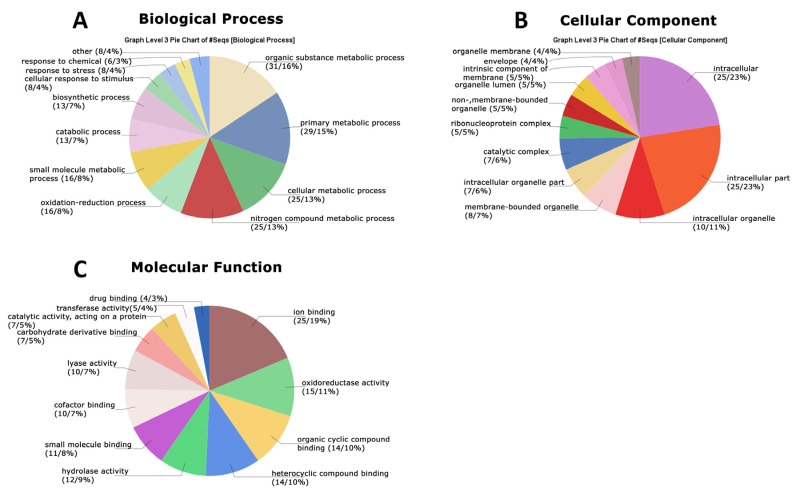
GO terms identified in protein secretome data at 12 days after inoculation (12 DAI) of *Panus lecomtei* considering two conditions: presence or absence of gossypol in cottonseed.

**Figure 8:**
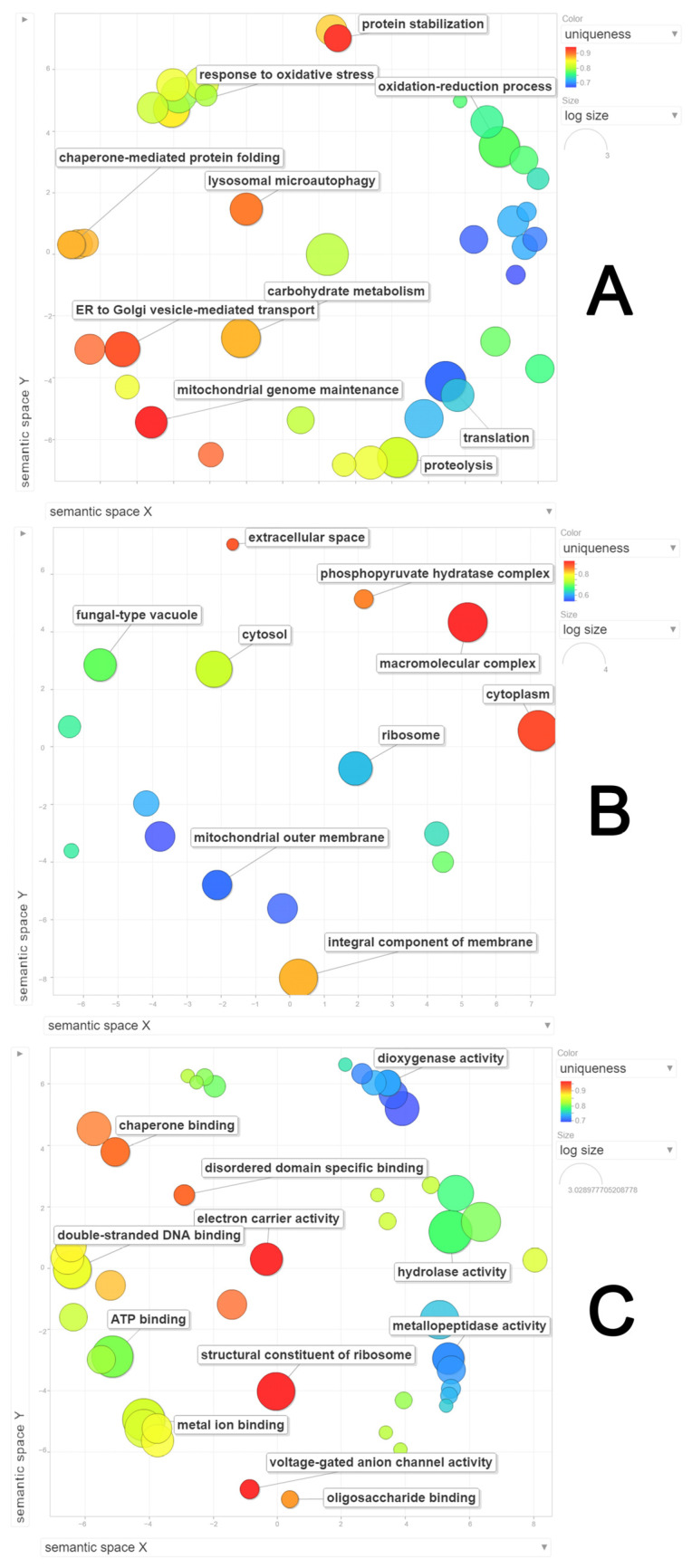
Semantic clustering of proteins identified at 12 days after inoculation (12 DAI) of *Panus lecomtei* considering two conditions: presence or absence of gossypol in cottonseed. (**A**) Biological process; (**B**) Cellular component; (**C**) Molecular function.

At 12 DAI, 12 biological process terms were observed, with the first four categories as observed at 6 DAI: organic substance metabolic process (16%), primary metabolic process (15%), cellular metabolic process (13%), and nitrogen-compound metabolic process (13%), followed by oxidation–reduction (8%), small molecule metabolic process (8%), catabolic process (7%), biosynthetic process (7%), and cellular response to stimulus (4%) (Figure 7A). Terms such as response to stress, response to chemical, and others were only observed at 12 DAI. The semantic grouping of proteins in this category showed a correlation between absolute frequency and grouping with similar terms, including response to oxidative stress, oxidation–reduction process, proteolysis, chaperone-mediated protein folding, translation, protein stabilization, and ER-to-Golgi-vesicle-mediated transport (Figure 8A). For cellular components, 12 terms were identified, of which 8 were also found at 6 DAI: intracellular (23%), intracellular part (23%), intracellular organelle (10%), membrane-bounded organelle (7%), intracellular organelle part (6%), catalytic complex (6%), ribonucleoprotein complex (5%), and nonmembrane-bounded organelle (5%) (Figure 7B). Terms such as organelle lumen, intrinsic component of the membrane, envelope, and organelle membrane were only identified at 12 DAI. The semantic grouping showed nine terms with a higher frequency, including macromolecular complex, cytoplasm, integral component of the membrane, cytosol, fungal-type vacuole, and ribosome (Figure 8B). For molecular functions, 12 categories were identified: ion binding (19%), oxidoreductase activity (11%), organic cyclic compound binding (10%), heterocyclic compound binding (10%), hydrolase activity (9%), small molecule binding (8%), cofactor binding (7%), lyase activity (5%), carbohydrate derivative binding (5%), catalytic activity acting on a protein (5%), transferase activity (4%), and drug binding (3%). Lyase activity was only identified at 12 DAI (Figure 7C). The semantic grouping showed that functions related to dioxygenase activity, metallopeptidase activity, metal ion binding, hydrolase activity, double-stranded DNA binding, and chaperone binding correlated with both absolute frequency and grouping with similar terms. Terms with the highest absolute frequency included structural constituent of the ribosome, electron carrier activity, and disordered domain-specific binding (Figure 8C).

**Figure 9:**
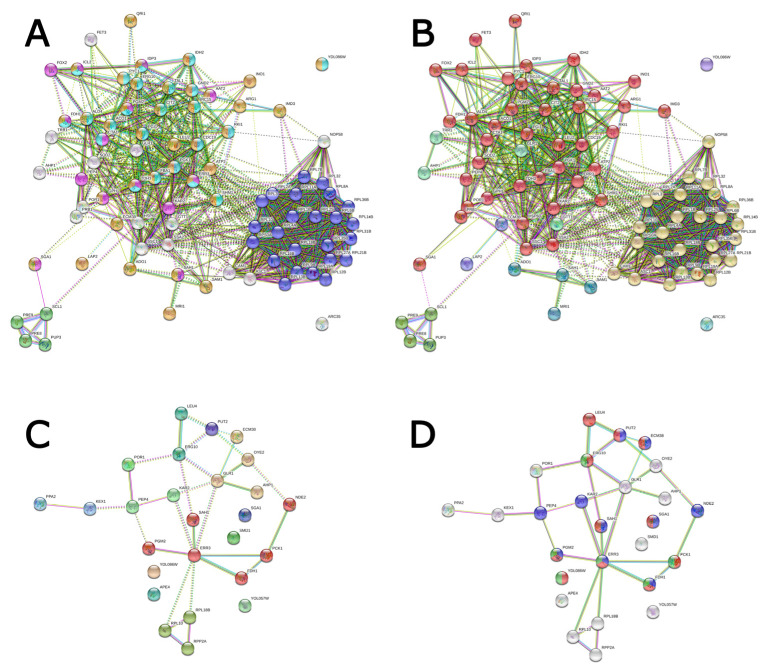
String network of regulated proteins of *Panus lecomtei* considering two conditions: presence or absence of gossypol in cottonseed. (**A**): Highlighting of regulated proteins associated with significantly overrepresented GO terms and pathways at 6 DAI (days after inoculation); (**B**): MCL clustering according to interactions network at 6 DAI; (**C**): highlighting of regulated proteins associated with significantly overrepresented GO terms and pathways at 12 DAI; and (**D**): MCL clustering according to interactions network at 12 DAI.

String analysis of differentially abundant proteins revealed a total of six interaction clusters at 6 DAI (Figure 9), four of which presented strong interaction networks and remarkable overlapping with enriched biological processes and pathways. Figure 7B shows the GO term Cytoplasmic Translation (FDR = 3.48 × 10^−14^) in blue, overlapped to the most intense cluster. Other biological processes overlapping to significantly clustered proteins comprised Proteasomal Ubiquitin-Independent Protein Catabolic Process (FDR = 0.0166) and Single-Organism Catabolic Process (FDR = 0.0325). Reinforcing the idea of metabolic regulation enhancement at this stage, KEGG pathways overlapping to interaction clusters comprised Microbial Metabolism in Diverse Environments (FDR = 2.68 × 10^−17^) and Metabolic Pathways (FDR = 1.46 × 10^−14^).

KEGG analysis of proteins exhibiting statistically significant differential abundance revealed an activation of 26 distinct pathways at 6 DAI. These pathways are primarily associated with carbohydrate metabolism, amino acid metabolism, and the biodegradation and metabolism of xenobiotics. Notably, certain pathways were exclusively detected at 6 DAI, including fructose and mannose metabolism, glycine, serine, and threonine metabolism, xenobiotic biodegradation by cytochrome P450, and drug metabolism by cytochrome P450. At 12 DAI, only seven distinct pathways were activated. Similar to results observed at 6 DAI, these pathways are involved in carbohydrate metabolism, amino acid metabolism, and biodegradation and metabolism of xenobiotics.

### 3.5. Partial Purification of Laccase Enzyme Fractions and Evaluation of Free-Gossypol Degradation

Data showed that *P. lecomtei* BRM044603 efficiently degraded FG during cultivation, with secretion of oxidative and proteolytic enzymes active during this process. To identify specific extracellular enzymes involved in FG degradation, a partial purification of crude enzymatic extracts was performed from culture samples under the two analysed conditions. Samples were subjected to size-exclusion chromatography using an S-200 column, resulting in distinct protein profiles. For the protein profile of *P. lecomtei* BRM044603 cultivated in the presence of gossypol, a total of nine fractions were obtained, with seven fractions obtained in the absence of gossypol. All fractions were quantified for total protein content (measured at A280 and with the Bradford assay) and for laccase enzyme activity. Among the fractions following cultivation in the presence of gossypol, only fractions S2 and S3 exhibited laccase enzyme activity (Supplementary Table S1). All obtained fractions were subject to synthetic gossypol degradation assays for 60 min at 240 rpm. Among these fractions, five were capable of degrading gossypol: S1, S2, S3, S4, and S8, although degradation to trace levels was observed only in the fractions with laccase activity (S2 and S3) (Supplementary Figure S2). Degradation analyses of the fractions obtained following cultivation without gossypol also revealed three positive fractions capable of gossypol degradation (Supplementary Figure S2). Similarly, synthetic gossypol degradation to trace levels was achieved only in the fraction displaying laccase activity (S3).

## 4. Discussion

The biodegradation of gossypol by fungi offers potential for the bioeconomy, where degradation specificity can be combined with enhancement of the nutritional value of residues, with fungal growth enriching with proteins and bioactive compounds such as ergosterol and β-glucans. Not all fungi are able to degrade gossypol in cottonseed. According to [58], for example, out of a total of 58 screened fungal isolates, only 8 were effective in degradation of gossypol, with the greatest degradation (65.2%) observed for *Fusarium thapsinum*, and the lowest degradation (30.4%) for *Fusarium chlamydospora*. Such data highlight that fungi from the same genus may exhibit differences in degradation efficiency. Here, *P. lecomtei* BRM044603 showed considerable efficacy in degradation of gossypol, eliminating 70% of FG in cottonseed. Data from identical bioassays conducted previously by the group revealed that this macrofungus also increases crude protein content in cottonseed by 37.6% [34], relevant for animal supplementation.

Data here revealed that degradation initiated at an early stage during the cultivation period, with the rate increasing over time, resulting in 70% degradation after 15 days of cultivation. Such a trend for increased degradation over time was also observed for the macrofungus *Pleurotus florida* [59], as well as for *Candida tropicalis* [60], where investigation of variables of initial moisture content, inoculum concentration, initial pH, and incubation temperature all showed that cultivation time had the greatest influence on gossypol degradation rate. With a correlation, therefore, between fungal mycelial growth on the substrate and FG degradation, it appears that degradation of gossypol requires an increase in microbial biomass, which is influenced by time, for any particular microorganism employed in the process. This also suggests that microorganisms do not initially direct their metabolism towards gossypol degradation, potentially indicating that this secondary metabolite does not affect the growth of such resistant or tolerant microorganisms. This hypothesis is supported with enzymatic activity data in this study, where unaltered enzymatic patterns (except for laccases) were observed in *P. lecomtei* cultivated both in the presence or absence of gossypol. Similar responses to the presence or absence of gossypol have also been observed in other fungi, such as *Aspergillus* sp., which, when grown with gossypol as the sole carbon source, was shown to exhibit a growth curve similar to that when cultivated in a glucose medium [61].

Biodiversity, which is a key component of the bioeconomy, can be defined as the variety and complexity among living organisms, encompassing species, DNA, genes, proteomes, metabolomes, and their interactions with ecological systems [62,63]. In this context, the biotechnological application of *P. lecomtei*, combined with omics tools such as proteomics, is appropriate for the identification and preservation of biodiversity. Whilst proteomics has been employed in a number of studies for the identification of proteins involved in detoxification processes [64,65,66,67,68], the proteomic data obtained here are unique both in relation to the macrofungus *P. lecomtei* as well as with regard to the degradation of gossypol in the lignocellulosic substrate.

PCA data and relative protein abundance profiles indicated a clear distinction in metabolic response in *P. lecomtei* BRM044603 following growth on CWCS and CWCT (Supplementary Figure S1). Global GO and semantic clustering data indicate that the fungal response also undergoes alterations at 6 DAI and 12 DAI, as observed in the biological process categories. In general, GO biological process terms at 6 DAI indicated functions related to constitutive metabolism, such as organic substance metabolic process, primary metabolic process, cellular metabolic process, nitrogen-compound metabolic process, small-molecule metabolic process, and catabolic process. String analysis also revealed a metabolic response of *P. lecomtei* to gossypol at 6 DAI related to protein metabolism, where enrichment of translation, proteasome catabolism and other catabolic processes were observed. At 12 DAI, by contrast, terms related to response to stress, response to chemical, and others were identified. Semantic clustering data also highlighted a distinct response at the two analysed time points. At 6 DAI, categories related to glucose metabolism, protein expression, and oxidative stress were observed. At 12 DAI, in addition to the categories identified at 6 DAI, a more intense response to oxidative stress was observed, with categories related to protein protection and maintenance of mitochondrial DNA identified.

Toxicity of gossypol in animals is associated with reduced antioxidant levels and increased pro-oxidant formation due to interactions with biological membranes that promote the generation of reactive oxygen species [69,70]. The proteomic data for *P. lecomtei* suggest that an adaptive response to oxidative stress over time is important in fungal degradation of gossypol. This is corroborated by previous studies, such as that by [71], who reported an increase in the antioxidant enzymes catalase and glutathione reductase during the consortial growth of *Aspergillus niger*, *Trichoderma reesei*, and *Phanerochaete chrysosporium* on gossypol as a carbon source.

The enzymatic and proteomic data presented here suggest that both the catabolism and anabolism of the fungus are directed towards survival on the lignocellulosic substrate, and that the presence of toxic gossypol does not, initially, interfere with the response of *P. lecomtei*. GO analysis of proteomic data at 12 DAI on CWCS indicated a response to oxidative stress, with the heat shock proteins also among the abundant proteins. This later time point was chosen for investigation as it precedes the peak of gossypol degradation. The data suggest, therefore, that in response to increased oxidative stress, *P. lecomtei* enhances the degradation of gossypol. This hypothesis is further supported with an analysis of VIP scores, where the protein laccase is more abundant at 12 DAI in the presence of gossypol. As shown in Supplementary Figure S2, this enzyme was shown to directly participate in gossypol degradation. Laccase enzymes from different microorganisms have previously been reported to play a significant role in the degradation of this substance [59,72,73,74,75,76,77], resulting from oxidative–reduction processes catalysed by the enzyme during lignolysis, synergizing with substrate colonization and subsequent fungal growth.

At 6 DAI, although laccase activities in the fungus were not abundant when grown in the presence of gossypol, the cytochrome P450 complex and the auxiliar cytochrome b5 oxidative enzyme were identified at this time point. This latter protein is known to play a role in modulation of cytochrome P450 [78], with these two monooxygenases extensively investigated and directly involved in drug metabolism and degradation of xenobiotic compounds [79,80,81,82]. Given this, the data suggest that, in addition to a distinct metabolic response, *P. lecomtei* also expresses different oxidative enzymes involved in gossypol degradation at different growth stages.

## 5. Conclusions

In conclusion, the biotreatment of cottonseed by *P. lecomtei* BRM044603 provides an efficient method for gossypol degradation in this residue that is appropriate for animal supplementation. A complex adaptive response to FG was observed in the fungus, with an emphasis on energy production for growth and survival, together with an activation of oxidative enzymes. The complete degradation of synthetic gossypol observed with laccase enzyme fractions, together with evidence for their constitutive secretion, irrespective of the presence of FG, highlight the potential for application of this fungus in industrial applications and bioremediation. Laccase enzymes are attractive due to their simple catalytic principles (substrate and O_2_ presence), apparent stability, and lack of inhibition. These enzymes can oxidize a wide range of organic and inorganic substrates, including mono-, di-, polyphenols, aminophenols, methoxyphenols, and metal complexes, making them versatile for numerous biotechnological applications [83], including bioremediation and detoxification of aromatic pollutants [84,85]. Continued investigation of this degradation model, exploring additional fungi and important renewable sources, will contribute to biodiversity preservation and promote sustainability in the production of valuable resources.

## Data Availability

Data are available within the article and Supplementary Materials.

## Supplementary Materials

The following supplementary information can be downloaded at: https://www.mdpi.com/article/10.3390/jof10050321/s1, Supplementary Figure S1: A—Profiles of relative protein abundance of *Panus lecomtei* grouped at 6 DAI; PLS-DA 3D showing clustering of replicates and differentiation between conditions at 6 DAI (0 = CWCS [presence of gossypol] and 1 = CWCT [absence of gossypol]); C—Profiles of relative protein abundance of *Panus lecomtei* grouped at 12 DAI; D-PLS-DA 3D showing clustering of replicates and differentiation between conditions at 12 DAI (0 = CWCS [presence of gossypol] and 1 = CWCT [absence of gossypol]). Supplementary Figure S2. Partially purified enzymatic extracts of *Panus lecomtei* obtained from cultivation in crushed whole cottonseed (CWCS) and chemically treated crushed whole cottonseed (CWCT) for synthetic gossypol degradation. Supplementary Table S1. Purification analysis of the laccase enzyme using molecular exclusion chromatography of fractions obtained from the cultivation of *Panus lecomtei* CC 40 for 12 days in untreated crushed whole cottonseed containing gossypol (CWCS) and crushed whole cottonseed chemically treated (CWCT) with 2% Ca(OH)_2._ S = After Superdex S-200.
