# Supplementary material for: An Enzymatic and Proteomic Analysis of Panus lecomtei during Biodegradation of Gossypol in Cottonseed
Source: J Fungi (Basel). 2024 Apr 27;10(5):321. doi: 10.3390/jof10050321 (PMC11121953; doi:10.3390/jof10050321)
Supplement: Supplementary file 1 [file jof-10-00321-s001.zip › Supplementary Table S1.pdf]

Supplementary Table S1. Purification analysis of the laccase enzyme using molecular exclusion chromatography of fractions obtained from the cultivation of *Panus lecomtei* CC 40 for 12 days in untreated crushed whole cottonseed-containing gossypol (CWCS) and crushed whole cottonseed chemically treated (CWCT) with 2% $Ca(OH)_2$. S= After Superdex S-200.

**Fraction**	**Volume (ml)**	**Protein (mg/ml)**	**Enzyme Activity (U/ml)**
CWCS-Crude extract	10	5,28	316,68
CWCS-S1	2	0,15	0,00
CWCS-S2	2	0,30	16,67
CWCS-S3	2	1,15	27,78
CWCS-S4	2	0,12	0,00
CWCS-S5	2	0,96	0,00
CWCS-S6	2	0,88	0,00
CWCS-S7	2	0,85	0,00
CWCS-S8	2	0,65	0,00
CWCS-S9	2	0,63	0,00
CWCT-Crude extract	10	2,34	51,08
CWCT-S2	2	0,11	16,67
CWCT-S3	2	0,30	0,00
CWCT-S4	2	0,12	0,00
CWCT-S5	2	0,92	0,00
CWCT-S6	2	0,91	0,00
CWCT-S7	2	0,79	0,00
CWCT-S8	2	0,83	0,00
