# Supplementary figures and images for: An Enzymatic and Proteomic Analysis of Panus lecomtei during Biodegradation of Gossypol in Cottonseed
Source: J Fungi (Basel). 2024 Apr 27;10(5):321. doi: 10.3390/jof10050321 (PMC11121953; doi:10.3390/jof10050321)

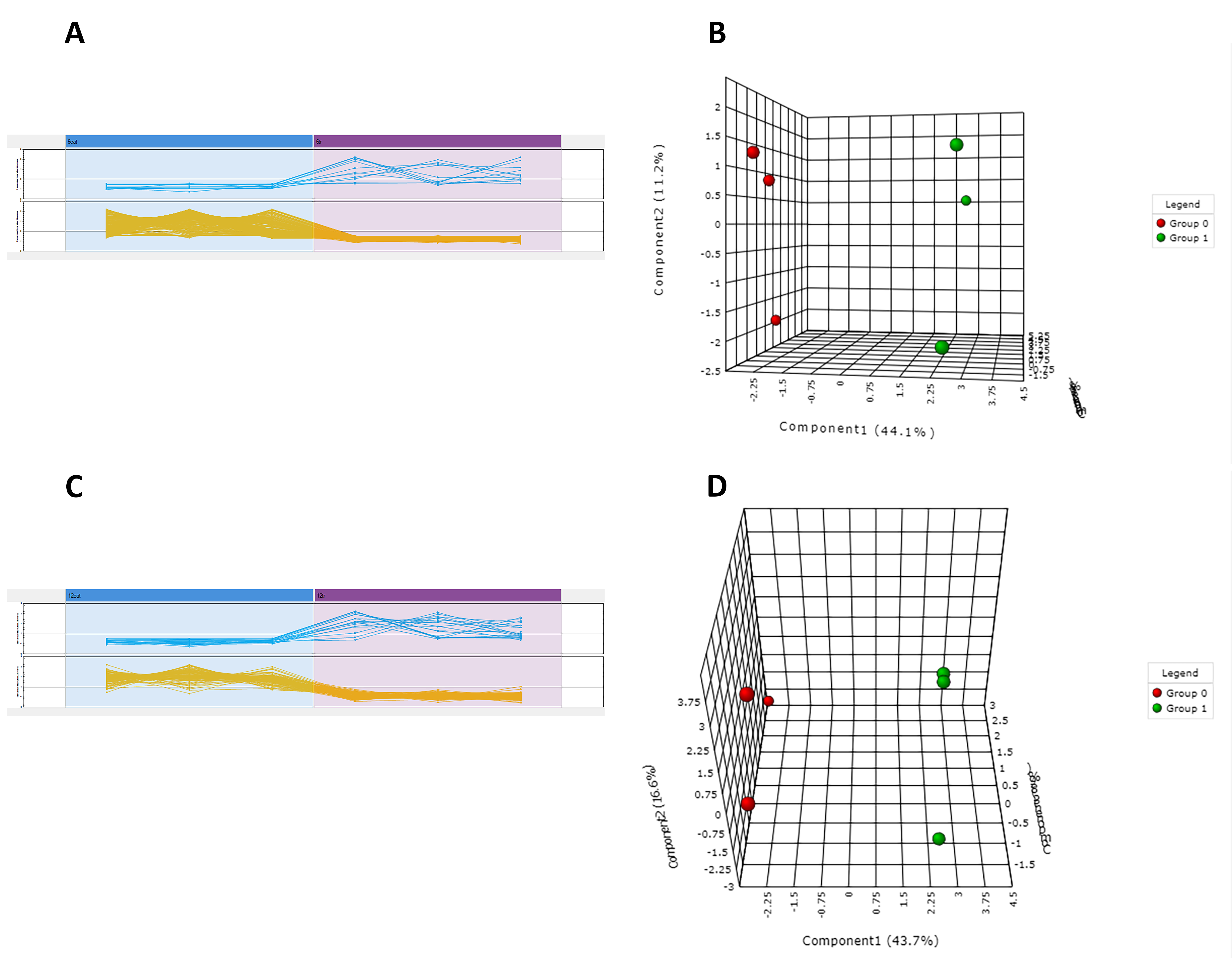

Supplement: Supplementary file 1 [file jof-10-00321-s001.zip › Supplementary Figure S1.tif]
